# Comparative Efficacy and Safety of Tegoprazan Versus Proton Pump Inhibitors for Erosive Esophagitis: A Systematic Review and Meta-Analysis

**DOI:** 10.7759/cureus.83302

**Published:** 2025-05-01

**Authors:** Helai Hussaini, Tamiru M Kebede, Tekleweyn S Afework, Deepa Kumari, Olaniyi Fadeyi, Sandipkumar S Chaudhari, Ihtisham Habib, Shamsha Hirani

**Affiliations:** 1 Internal Medicine, West Anaheim Medical Center, Anaheim, USA; 2 General Medicine, Jimma University Public Science and Medical School, Jimma, ETH; 3 General Surgery, St. Paul's Hospital Millennium Medical College, Addis Ababa, ETH; 4 Internal Medicine, St. Paul's Hospital Millennium Medical College, Addis Ababa, ETH; 5 Medicine, Bahria University Medical and Dental College, Karachi, PAK; 6 Cardiothoracic Surgery, University of Alabama at Birmingham, Birmingham, USA; 7 Family Medicine, University of North Dakota School of Medicine and Health Sciences, Fargo, USA; 8 Internal Medicine, Lady Reading Hospital Medical Teaching Institute, Peshawar, PAK; 9 Cardiology, Baqai Hospital, Karachi, PAK

**Keywords:** efficacy, esophagitis, meta-analysis, safety, tegoprazan

## Abstract

This systematic review and meta-analysis evaluated the efficacy and safety of tegoprazan compared to proton pump inhibitors (PPIs) in the treatment of erosive esophagitis (EE). A comprehensive literature search was conducted across multiple electronic databases from inception to 25 March 2025. Randomized controlled trials comparing tegoprazan with PPIs in patients with endoscopically confirmed EE were included. Primary outcomes were healing rates and safety profiles. Four randomized controlled trials comprising 963 patients were included. Meta-analysis revealed comparable healing rates between tegoprazan and PPIs (RR: 1.03, 95% CI: 0.97-1.10), with no significant differences observed at both four weeks (RR: 1.05, 95% CI: 0.96-1.16) and eight weeks (RR: 1.01, 95% CI: 0.96-1.06). Safety analyses demonstrated similar profiles between treatments, with no significant differences in treatment-emergent adverse events (RR: 0.93, 95% CI: 0.73-1.35), drug-related adverse events (RR: 0.93, 95% CI: 0.51-1.69), or serious adverse events (RR: 1.07, 95% CI: 0.12-9.29). Symptom relief was comparable between groups across all studies, with tegoprazan showing consistent efficacy, regardless of CYP2C19 genotype. Most patients had mild to moderate EE (LA grades A and B), limiting conclusions about efficacy in severe cases. This first meta-analysis directly comparing tegoprazan with PPIs suggests that tegoprazan is an effective and safe alternative to PPIs for EE treatment. Its efficacy independent of CYP2C19 polymorphism represents a potential advantage. However, limitations include the small number of studies, predominantly Asian populations, and relatively short follow-up periods. Further research is needed to assess long-term outcomes and efficacy in diverse populations.

## Introduction and background

Erosive esophagitis (EE), a more severe form of gastroesophageal reflux disease (GERD), is characterized by mucosal breaks in the esophagus due to prolonged exposure to gastric acid [1]. It affects millions of individuals globally, significantly impairing quality of life and increasing the risk of complications such as esophageal strictures and Barrett’s esophagus [2]. The primary therapeutic goal in EE is to promote mucosal healing, relieve symptoms, and prevent recurrence, for which acid-suppressive therapy remains the cornerstone of management [3].

For decades, proton pump inhibitors (PPIs), such as omeprazole, esomeprazole, and lansoprazole, have been the mainstay treatment for EE, owing to their efficacy in suppressing gastric acid secretion [4]. However, PPIs exhibit several limitations, including a relatively slow onset of action, variable effectiveness depending on CYP2C19 metabolism, incomplete control of nocturnal acid breakthrough, and potential long-term safety concerns, such as increased risk of fractures, renal impairment, infections, and micronutrient deficiencies [5,6]. These shortcomings have driven the search for novel acid-suppressive agents with more favorable pharmacological profiles.

Tegoprazan is a next-generation potassium-competitive acid blocker (P-CAB) that inhibits the H⁺/K⁺-ATPase enzyme in a reversible and potassium-competitive manner. Unlike PPIs, tegoprazan does not require acid activation and demonstrates a faster onset of action, more consistent acid suppression, regardless of CYP2C19 polymorphism, and sustained efficacy over 24 hours [7-9].

Recent randomized controlled trials (RCTs) have explored Tegoprazan’s efficacy and safety in the treatment of EE, reporting non-inferior or even superior healing rates compared to traditional PPIs, as well as comparable safety profiles [10,11]. However, results across individual studies have been inconsistent and limited by small sample sizes. Given the clinical importance of identifying optimal therapy for EE and the emerging role of tegoprazan as a potential alternative to PPIs, there is a critical need to synthesize current evidence to better inform clinical decision-making. To our knowledge, no comprehensive meta-analysis has yet been conducted to systematically compare the efficacy and safety of tegoprazan versus PPIs in the treatment of EE. Therefore, the objective of this systematic review and meta-analysis is to evaluate and compare the effectiveness and safety of tegoprazan and PPIs in adult patients with EE.

## Review

Methodology 

*Literature Search and Search Strategy* 

A comprehensive literature search was conducted in multiple electronic databases (TK and TS), including PubMed, Embase, Cochrane Library, Web of Science, and clinical trial registries, from inception to 25 March 2025. The search strategy employed a combination of Medical Subject Headings (MeSH) terms and free-text keywords related to "tegoprazan", "potassium-competitive acid blocker", "P-CAB", "proton pump inhibitor", "PPI", "erosive esophagitis", and "gastroesophageal reflux disease". Details of the search strategy are given in the Appendix. Additionally, reference lists of relevant reviews and included studies were manually screened to identify potential studies not captured by the electronic search. No language restrictions were applied. Search was performed by two authors independently (TK and TA). Any disagreement between the two authors was resolved through discussion.

Study Selection 

Two independent reviewers screened titles and abstracts of retrieved articles to identify potentially eligible studies (DK and OF). Full texts of relevant articles were then evaluated against the inclusion criteria. Studies were eligible if they (1) were randomized controlled trials (RCTs); (2) compared tegoprazan with PPIs in patients with endoscopically confirmed EE; (3) reported clinical outcomes, including endoscopic healing rates, symptom improvement, or safety outcomes; and (4) had a minimum follow-up duration of two weeks. Non-randomized studies, case reports, editorials, review articles, and abstracts without full texts were excluded. Disagreements between reviewers were resolved through discussion or by consulting a third reviewer.

Data Extraction and Outcomes 

Data extraction was performed independently by two reviewers using a standardized data collection form (SC and IH). The following information was extracted: first author, publication year, study design, sample size, patient demographics, intervention details, control groups, and outcomes. The efficacy outcome assessed in this study was the healing rate (as defined by the included studies). Safety outcomes included treatment-emergent adverse events (TEAE), serious TEAE, and drug-related TEAE. Data extraction was performed by two authors. Any disagreement between the two authors was resolved through discussion.

Quality Assessment 

To evaluate the methodological rigor of the studies included, the Cochrane Risk of Bias Tool 2.0 was employed. This tool examines potential bias across five key areas: the randomization procedure, adherence to assigned interventions, completeness of outcome data, accuracy of outcome measurement, and reporting selectivity. Each area was rated as presenting a "low risk," "some concerns," or a "high risk" of bias. The assessment was independently conducted by two reviewers (DK and IH), with any discrepancies addressed through discussion or, if necessary, by involving a third reviewer.

Data Analysis 

Data analysis was performed using RevMan (Cochrane Collaboration, London, UK) software. For each outcome, including healing rates and safety measures, risk ratios (RRs) were calculated alongside 95% confidence intervals (CIs). A p-value below 0.05 was considered statistically significant. A random-effects model was applied to synthesize results across studies and generate overall estimates. To assess variability among studies, the I² statistic was used. An I² value under 50% was interpreted as indicating low heterogeneity, while values exceeding 50% suggested a considerable degree of heterogeneity.

Results 

**Figure 1:**
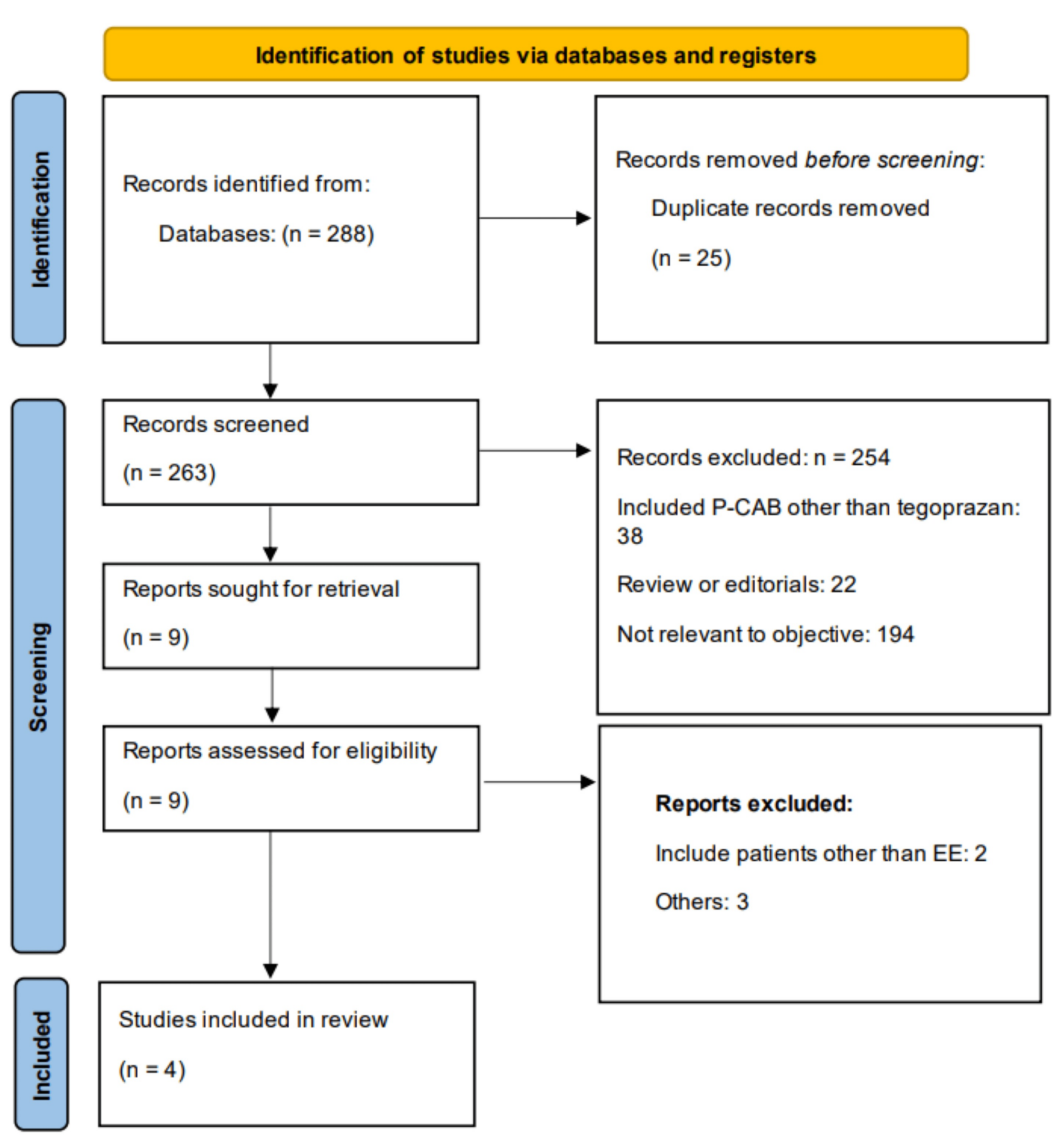
PRISMA flowchart (study selection process) PRISMA: Preferred Reporting Items for Systematic Reviews and Meta-Analyses

**Figure 2:**
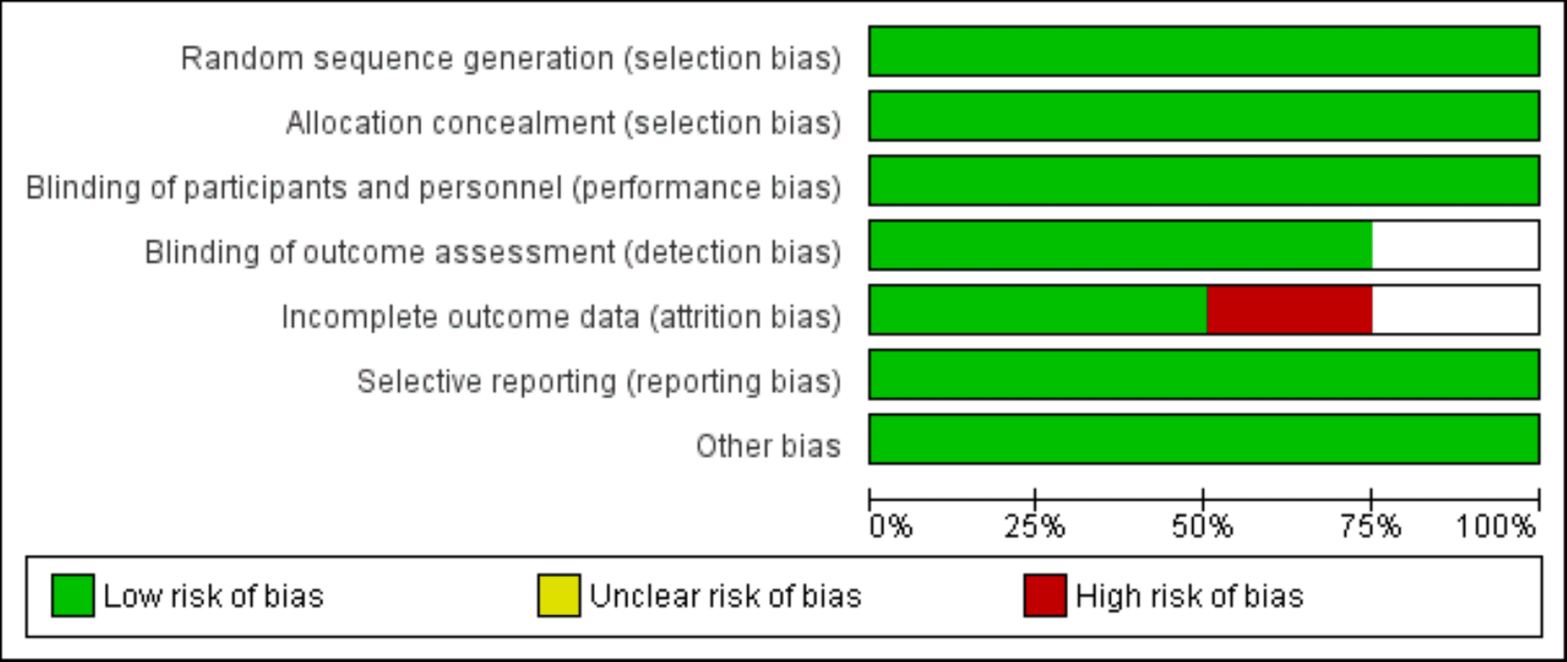
Risk of bias graph

Online database searching yielded 288 studies. After removing 25 duplicates, 263 records were initially screened. Full-texts of nine studies were obtained, and five studies did not fulfill the eligibility criteria. Finally, four studies were included in the meta-analysis. The study selection process is shown in Figure 1. The four RCTs randomized a total of 963 adult patients with healed EE to either tegoprazan or PPI. Three studies recruited subjects from Korea, while one study was conducted in China. The duration of studies ranged from four weeks to 24 weeks. The randomized subjects had a mean age between 45.9 and 55.99 years. Table 1 presents the characteristics of the included studies. Figure 2 presents the risk of bias graph.

**Table 1:** Characteristics of the studies RCT: Randomized controlled trial

Author and Year	Study Design	Country	Groups	Sample Size	Dose	Follow-up	Male (n)	Age (Years)	Grade C/D
Cho et al., 2022 [12]	Phase III RCT	Korea	Tegoprazan	154	25 mg	24 Weeks	114	55.4	8
			Lansoprazole	151	15 mg		110	55.99	10
Lee et al., 2019 [11]	Phase III RCT	Korea	Tegoprazan	99	50 mg	8 Weeks	62	52.7	4
			Esomeprazole	99	40 mg		53	50.4	4
Shin et al., 2025 [13]	Phase IV RCT	Korea	Tegoprazan	103	50 mg	4 Weeks	61	53.97	9
			Lansoprazole	109	30 mg		75	52.06	10
Zhu et al., 2024 [14]	Phase III RCT	China	Tegoprazan	123	50 mg	8 Weeks	90	48	11
			Esomeprazole	125	40 mg		92	45.9	10

Rate of Healing

**Figure 3:**
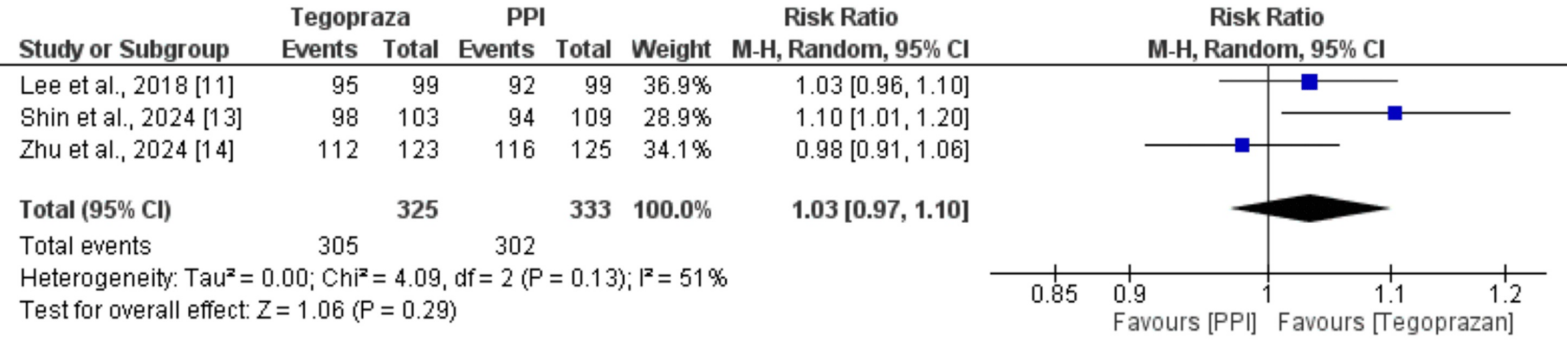
Comparing the healing rates between the two groups Sources: References [11,13,14]

Three studies compared the rate of healing between subjects who received tegoprazan and PPI, and the results are presented in Figure 3. Compared to PPI, tegoprazan has a similar rate of healing in subjects with EE, as no significant difference was reported between the two groups in terms of rate of healing (RR: 1.03, 95% CI: 0.97-1.10). Significant heterogeneity was reported among the study results (I-Square: 51%). 

One study followed subjects for four weeks and two studies for eight weeks. To understand the cause of heterogeneity, we performed pooled estimates for four weeks and eight weeks separately, and the results are presented in Table 2. There was no significant difference between tegoprazan and PPI in relation to four-week healing rate (RR: 1.05, 95% CI: 0.96-1.16) and eight-week healing rate (RR: 1.01, 95% CI: 0.96-1.06). No significant heterogeneity was reported in any of the subgroups (I² <25 % for both four-week and eight-week analyses). These findings suggest that treatment duration does not significantly influence the relative efficacy of tegoprazan compared to PPIs, with both medications demonstrating similar healing rates at both time points.

**Table 2:** Subgroup analysis of the healing rate RR: Risk ratio; CI: Confidence interval

Outcome	Subgroups	RR (95% CI)	I-Square
Healing Rate	Four weeks	1.05 (0.96-1.16)	25%
	Eight weeks	1.01 (0.96-1.06)	3%

Safety Outcomes 

Table 3 presents the results of safety outcomes. In terms of safety outcomes, our meta-analysis revealed comparable safety profiles between tegoprazan and PPIs. There was no significant difference in the risk of experiencing any TEAEs between the two treatment groups (RR: 0.93, 95% CI: 0.73-1.35, p=0.96). Similarly, the incidence of drug-related TEAEs was not significantly different between tegoprazan and PPI groups (RR: 0.93, 95% CI: 0.51-1.69, p=0.81). The occurrence of serious TEAEs was also comparable between the two treatments (RR: 1.07, 95% CI: 0.12-9.29, p=0.95). These findings indicate that tegoprazan demonstrates a safety profile similar to that of PPIs, with no significant increase in adverse events.

**Table 3:** Safety outcome analysis RR: Risk ratio; CI: Confidence interval; TEAE: Treatment-emergent adverse events

Safety Outcomes	RR (95% CI)	P-value
Any TEAE	0.93 (0.73-1.35)	0.96
Drug related TEAE	0.93 (0.51-1.69)	0.81
Serious TEAE	1.07 (0.12-9.29)	0.95

Symptoms Relief 

In all three studies, tegoprazan demonstrated non-inferior efficacy compared to PPIs (esomeprazole or lansoprazole) in terms of symptom improvement. Lee et al. [11] found no significant differences in the Reflux Disease Questionnaire (RDQ) total score changes between the tegoprazan and esomeprazole groups. Similarly, Shin et al. [13] reported comparable changes in RDQ and GERD Health-Related Quality of Life (GERD-HRQL) scores between tegoprazan and lansoprazole. Zhu et al. [14] observed similar improvements in RDQ total scores and GERD-HRQL in both tegoprazan and esomeprazole groups, with no statistically significant differences.

Regarding daily symptom diaries, all studies showed similar percentages of symptom-free days between the tegoprazan and PPI groups. In Shin et al.'s [13] study, tegoprazan demonstrated slightly higher proportions of heartburn-free days compared to lansoprazole, particularly in the early treatment phase, though this difference did not reach statistical significance. Lee et al.'s [11] study reported that reductions in regurgitation severity and frequency were statistically higher in the tegoprazan group at weeks four and eight compared to esomeprazole, but this difference disappeared after baseline adjustment. Zhu et al. [14] found no significant differences in daytime or nighttime symptom resolution rates.

Discussion 

The following is a summary of the main findings from the current review and meta-analysis. Firstly, the healing rate was similar between patients who received tegoprazan and those treated with PPIs. Secondly, in the safety analysis, the incidence of TEAEs, drug-related TEAEs, and serious TEAEs did not differ significantly between the two groups. Additionally, the majority of patients in both arms presented with mild-to-moderate EE (LA grades A and B), while severe cases (LA grades C and D) were relatively uncommon. These findings suggest that tegoprazan offers comparable efficacy and safety to PPIs in the treatment of EE. In the meta-analysis conducted by Cho et al. [15], including patients with Helicobacter pylori reported that H. pylori eradication and adverse drug event rates with tegoprazan and PPI-based treatments were similar. Another meta-analysis including all available P-CAB found that they are non-inferior to PPIs as therapy for patients with GERD. The safety outcomes for potassium-competitive acid inhibitors are similar to those for PPIs [16].

In July 2018, tegoprazan received approval in South Korea for the management of both EE and non-erosive reflux disease (NERD), marking it as the first clinically available potassium-competitive acid blocker (P-CAB) specifically for NERD treatment [17-18]. As a next-generation P-CAB, tegoprazan operates through a distinct mechanism compared to traditional PPIs [19]. It begins suppressing gastric acid swiftly following the first dose, and multiple preclinical and clinical investigations have confirmed its prolonged acid-inhibitory effects [20]. The results of this meta-analysis suggested that tegoprazan is a valid option for managing EE.

This is the first meta-analysis directly comparing tegoprazan and PPI in patients with EE. Venoprazan is one of the first P-CABS approved to be used in EE and meta-analysis, including six trials assessing the efficacy and safety of the drug in patients with GERD and EE. Vonoprazan demonstrated comparable efficacy and safety to PPIs in managing erosive GERD, and it outperformed PPIs, specifically in patients with more advanced EE [21]. However, due to the limited availability of data regarding the effectiveness of tegoprazan in treating severe EE, further research is necessary to strengthen the evidence in this specific subgroup.

The effectiveness of PPIs is significantly affected by genetic variations in the CYP2C19 enzyme, as this enzyme is primarily responsible for metabolizing most PPIs. However, tegoprazan at a 25 mg dose showed comparable rates of endoscopic remission across different CYP2C19 genetic profiles (p=0.7637) [12]. This can be attributed to the fact that tegoprazan and lansoprazole follow distinct primary metabolic pathways - CYP3A4 for tegoprazan and CYP2C19 for lansoprazole [22]. As a result, tegoprazan's therapeutic response is more consistent across individuals and less dependent on genetic differences. Moreover, it is less likely to interfere with medications metabolized by CYP2C19, such as clopidogrel. Given that symptom relief is a key goal in treating EE, earlier research has also compared symptom control between P-CABs and PPIs, revealing similar levels of effectiveness [19,23]. Consistently, in all included studies, similar percentages of symptom-free days were observed between the tegoprazan and PPI groups. Such results suggest that tegoprazan, like PPI, could effectively control the symptoms in patients.

Safety analysis included TEAE, drug-related TEAE, and serious TEAE, which were compared between tegoprazan and PPI. Pooled analysis showed that the safety profile between the two treatments was similar, as no significant difference was observed between the two groups in terms of TEAE, drug-related TEAE, and serious TEAE. The most commonly reported adverse events across studies included increased blood gastrin levels, gastrointestinal disorders, and mild liver function abnormalities, which were generally mild-to-moderate in severity and rarely led to treatment discontinuation. This shows that the safety profile of tegoprazan is comparable to that of PPI.

Our study has several limitations that should be considered when interpreting the results. First, the small number of included studies (n=4) limited our ability to perform robust meta-analyses for some outcomes and subgroup analyses, and only three studies have assessed the efficacy outcome (i.e., healing rate). Secondly, all included studies were conducted in Asian populations (South Korea and China), potentially limiting the generalizability of our results to other ethnicities and geographical regions. Third, the relatively short follow-up periods (four to eight weeks) preclude conclusions about the long-term efficacy and safety profiles of tegoprazan compared to PPIs, particularly regarding rare adverse events that might emerge with prolonged use. Future studies with larger sample sizes, more diverse populations, longer follow-up periods, and independent funding sources are needed to address these limitations.

## Conclusions

Tegoprazan demonstrated comparable efficacy and safety to PPIs in the treatment of erosive esophagitis. Meta-analysis of four RCTs revealed no significant differences in the healing rates at both four and eight weeks, with similar safety profiles between both treatment groups. The findings of previous studies, including network meta-analysis, showed non-inferiority of PCABs, including tegoprazan, in comparison to PPIs. These analyses suggest tegoprazan’s potential advantages in acid suppression duration and symptom relief. Larger multinational trials with extended observation periods are needed to clarify tegoprazan’s role in treatment algorithms, particularly regarding long-term maintenance therapy and comparative effectiveness against newer P-CABs.

## Appendices

Search Strategy for PubMed

(((((("Tegoprazan"[Supplementary Concept] OR "Tegoprazan"[Title/Abstract] OR "K-CAB"[Title/Abstract] OR "CJ-12420"[Title/Abstract] OR "RQ-00000004"[Title/Abstract] OR "CJ12420"[Title/Abstract])) OR (("Potassium Competitive Acid Blocker"[Title/Abstract] OR "Potassium-Competitive Acid Blocker"[Title/Abstract] OR "P-CAB"[Title/Abstract] OR "PCAB"[Title/Abstract] OR "P CAB"[Title/Abstract]))) AND ((("Proton Pump Inhibitors"[Mesh] OR "Proton Pump Inhibitor*"[Title/Abstract] OR "PPI"[Title/Abstract] OR "PPIs"[Title/Abstract] OR "Lansoprazole"[Mesh] OR "Lansoprazole"[Title/Abstract] OR "Omeprazole"[Mesh] OR "Omeprazole"[Title/Abstract] OR "Pantoprazole"[Mesh] OR "Pantoprazole"[Title/Abstract] OR "Rabeprazole"[Mesh] OR "Rabeprazole"[Title/Abstract] OR "Esomeprazole"[Me sh] OR "Esomeprazole"[Title/Abstract] OR "Dexlansoprazole"[Supplementary Concept] OR "Dexlansoprazole"[Title/Abstract])))) AND ((("Esophagitis"[Mesh] OR "Esophagitis"[Title/Abstract] OR "Oesophagitis"[Title/Abstract] OR "Erosive Esophagitis"[Title/Abstract] OR "Erosive Oesophagitis"[Title/Abstract]) OR ("Gastroesophageal Reflux"[Mesh] OR "Gastroesophageal Reflux"[Title/Abstract] OR "GERD"[Title/Abstract] OR "Gastro-oesophageal Reflux"[Title/Abstract] OR "Reflux Esophagitis"[Title/Abstract] OR "Reflux Disease"[Title/Abstract] OR "Reflux Oesophagitis"[Title/Abstract])))

Search Strategy for EMBASE

('tegoprazan'/exp OR 'tegoprazan':ti,ab,kw OR 'cj-12420':ti,ab,kw OR 'cj12420':ti,ab,kw OR 'k-cab':ti,ab,kw OR 'rq-00000004':ti,ab,kw) OR ('potassium competitive acid blocker'/exp OR 'potassium competitive acid blocker':ti,ab,kw OR 'potassium-competitive acid blocker':ti,ab,kw OR 'p-cab':ti,ab,kw OR 'pcab':ti,ab,kw OR 'p cab':ti,ab,kw) AND ('proton pump inhibitor'/exp OR 'proton pump inhibitor*':ti,ab,kw OR 'ppi':ti,ab,kw OR 'ppis':ti,ab,kw OR 'lansoprazole'/exp OR 'lansoprazole':ti,ab,kw OR 'omeprazole'/exp OR 'omeprazole':ti,ab,kw OR 'pantoprazole'/exp OR 'pantoprazole':ti,ab,kw OR 'rabeprazole'/exp OR 'rabeprazole':ti,ab,kw OR 'esomeprazole'/exp OR 'esomeprazole':ti,ab,kw OR 'dexlansoprazole'/exp OR 'dexlansoprazole':ti,ab,kw) AND ('esophagitis'/exp OR 'esophagitis':ti,ab,kw OR 'oesophagitis':ti,ab,kw OR 'erosive esophagitis':ti,ab,kw OR 'erosive oesophagitis':ti,ab,kw OR 'gastroesophageal reflux'/exp OR 'gastroesophageal reflux':ti,ab,kw OR 'gerd':ti,ab,kw OR 'gastro-oesophageal reflux':ti,ab,kw OR 'reflux esophagitis':ti,ab,kw OR 'reflux disease':ti,ab,kw OR 'reflux oesophagitis':ti,ab,kw) AND ('randomized controlled trial'/exp OR 'randomized controlled trial':ti,ab,kw OR 'controlled clinical trial'/exp OR 'controlled clinical trial':ti,ab,kw OR 'random*':ti,ab,kw OR 'trial':ti,ab,kw OR 'clinical trial'/exp OR 'clinical trial':ti,ab,kw)

Cochrane Library Search Strategy

#1 MeSH descriptor: [Proton Pump Inhibitors] explode all trees #2 (proton NEXT pump NEXT inhibitor*):ti,ab,kw OR (PPI):ti,ab,kw OR (PPIs):ti,ab,kw #3 MeSH descriptor: [Lansoprazole] explode all trees #4 (lansoprazole):ti,ab,kw #5 MeSH descriptor: [Omeprazole] explode all trees #6 (omeprazole):ti,ab,kw #7 MeSH descriptor: [Pantoprazole] explode all trees #8 (pantoprazole):ti,ab,kw #9 MeSH descriptor: [Rabeprazole] explode all trees #10 (rabeprazole):ti,ab,kw #11 MeSH descriptor: [Esomeprazole] explode all trees #12 (esomeprazole):ti,ab,kw #13 (dexlansoprazole):ti,ab,kw #14 #1 OR #2 OR #3 OR #4 OR #5 OR #6 OR #7 OR #8 OR #9 OR #10 OR #11 OR #12 OR #13 #15 (tegoprazan):ti,ab,kw OR (CJ-12420):ti,ab,kw OR (CJ12420):ti,ab,kw OR (K-CAB):ti,ab,kw OR (RQ-00000004):ti,ab,kw #16 (potassium NEXT competitive NEXT acid NEXT blocker):ti,ab,kw OR (potassium-competitive NEXT acid NEXT blocker):ti,ab,kw OR (P-CAB):ti,ab,kw OR (PCAB):ti,ab,kw OR (P NEXT CAB):ti,ab,kw #17 #15 OR #16 #18 MeSH descriptor: [Esophagitis] explode all trees #19 (esophagitis):ti,ab,kw OR (oesophagitis):ti,ab,kw OR (erosive NEXT esophagitis):ti,ab,kw OR (erosive NEXT oesophagitis):ti,ab,kw #20 MeSH descriptor: [Gastroesophageal Reflux] explode all trees #21 (gastroesophageal NEXT reflux):ti,ab,kw OR (GERD):ti,ab,kw OR (gastro-oesophageal NEXT reflux):ti,ab,kw OR (reflux NEXT esophagitis):ti,ab,kw OR (reflux NEXT disease):ti,ab,kw OR (reflux NEXT oesophagitis):ti,ab,kw #22 #18 OR #19 OR #20 OR #21 #23 #14 AND #17 AND #22 #24 MeSH descriptor: [Randomized Controlled Trial] explode all trees #25 (randomized):ti,ab,kw OR (randomised):ti,ab,kw OR (controlled):ti,ab,kw OR (trial):ti,ab,kw OR (clinical NEXT trial):ti,ab,kw #26 #24 OR #25 #27 #23 AND #26

Web of Science Search Strategy

TS=("Tegoprazan" OR "CJ-12420" OR "CJ12420" OR "K-CAB" OR "RQ-00000004" OR "Potassium Competitive Acid Blocker" OR "Potassium-Competitive Acid Blocker" OR "P-CAB" OR "PCAB" OR "P CAB") AND TS=("Proton Pump Inhibitor*" OR "PPI" OR "PPIs" OR "Lansoprazole" OR "Omeprazole" OR "Pantoprazole" OR "Rabeprazole" OR "Esomeprazole" OR "Dexlansoprazole") AND TS=("Esophagitis" OR "Oesophagitis" OR "Erosive Esophagitis" OR "Erosive Oesophagitis" OR "Gastroesophageal Reflux" OR "GERD" OR "Gastro-oesophageal Reflux" OR "Reflux Esophagitis" OR "Reflux Disease" OR "Reflux Oesophagitis") AND TS=("randomized controlled trial" OR "controlled clinical trial" OR "random*" OR "clinical trial")
